# Current IGFBP-Related Biomarker Research in Cardiovascular Disease—We Need More Structural and Functional Information in Clinical Studies

**DOI:** 10.3389/fendo.2018.00388

**Published:** 2018-07-16

**Authors:** Andreas Hoeflich, Robert David, Rikke Hjortebjerg

**Affiliations:** ^1^Department of Genome Biology, Leibniz Institute for Farm Animal Biology, Dummerstorf, Germany; ^2^Department of Cardiac Surgery, Reference and Translation Center for Cardiac Stem Cell Therapy, Rostock University Medical Center, Rostock, Germany; ^3^Department Life, Light and Matter, Interdisciplinary Faculty, Rostock University, Rostock, Germany; ^4^Medical Research Laboratory, Department of Clinical Medicine, Faculty of Health, Aarhus University, Aarhus, Denmark; ^5^The Danish Diabetes Academy, Odense, Denmark

**Keywords:** IGFBP, PAPP-A, IGFBP-fragment, mortality, cardiovascular diseases, IGF

## Abstract

Cardiovascular diseases are the leading cause of death around the world and the insulin-like growth factor (IGF)-system has multiple functions for the pathological conditions of atherosclerosis. IGF binding proteins (IGFBPs) are widely investigated as biomarkers for pathological disorders, including those of the heart. At the tissue level, IGFBP-1 to -6 decrease bioactivity of IGF-I and -II due to their high affinity IGF-binding sites. By contrast, in the circulation, the IGFBPs increase biological half-life of the IGFs and may therefore be regarded as positive regulators of IGF-effects. The IGFBPs may also exert IGF-independent functions inside or outside the cell. Importantly, the circulating IGFBP-concentrations are regulated by trophic, metabolic, and reproductive hormones. In a multitude of studies of healthy subjects and patients with coronary heart diseases, various significant associations between circulating IGFBP-levels and defined parameters have been reported. However, the complex hormonal and conditional control of IGFBPs may explain the lack of clear associations between IGFBPs and parameters of cardiac failure in broader studies including larger populations. Furthermore, the IGFBPs are subject to posttranslational modifications and proteolytic degradation by proteases, upon which the IGFs are released. In this review, we emphasize that, with the exception of IGFBP-4 and in sharp contrast to the preclinical studies, virtually all clinical studies do not have structural or functional information on their biomarker. The use of analytical systems with no discriminatory potential toward intact vs. fragmented IGFBPs represents a major issue in IGFBP-related biomarker research and an important focus point for the future. Overall, measurements of selected IGFBPs or more complex IGFBP-signatures of the family of IGFBPs have potential to identify pathophysiological alterations in the heart or patients with high cardiovascular risk, particularly if defined cohorts are to be assessed. However, a more thorough understanding of the dynamic IGF-IGFBP system as well as its proteases and protease inhibitors in both normal physiology and in cardiovascular diseases is necessary.

## Introduction

In Europe (1) and in the US (2), cardiovascular diseases are heading the statistics on causes of death. It is well-known that the insulin-like growth factor (IGF)-system actively contributes to the pathological conditions of atherosclerosis, including activation of smooth muscle cells and macrophages, angiogenesis, and restenosis (3). First of all, IGF-I and IGF-II are potent stimulators of smooth muscle cell proliferation (4, 5). In human arterial smooth muscle cells, IGF-I has been identified as a potent effector of chemotaxis (4). IGF-I has also been shown to increase the release of proinflammatory cytokines and low-density lipoprotein (LDL) uptake, which facilitate atherosclerosis and plaque instability (3). In addition, it is thought that the IGF-system supports accumulation of extracellular matrix in the vessel walls (3), which may occur by the control of matrix degrading enzymes (6). Under conditions of reduced IGF-I concentrations, levels of matrix proteins (actin and procollagen 3A1) are decreased, whereas matrix metalloproteinase levels (MMP-3 and−13) are elevated in smooth muscle cells (6). Supplementation by IGF-I normalizes both matrix proteins and matrix degrading enzymes (6). Interestingly, IGF-I concentrations in *in vitro* cultivated smooth muscle cells are affected when cell culture medium conditioned by macrophages is used.

IGF bioavailability is strictly regulated by six high-affinity IGF binding proteins (IGFBPs) that are ubiquitously produced in most tissues. The IGFBPs bind the IGFs on a 1:1 molar basis and prevent receptor activation, but they also serve to prolong IGF half-life. IGFBP-3 is the most abundant IGFBP in adult serum with a concentration of approximately 3,000 ng/mL, whereas the remaining IGFBPs circulate at concentrations of 20–500 ng/mL (7). Due to their affinities and high concentrations, <1% of IGF is circulating in the free form (7, 8). The acid labile subunit (ALS) is found almost exclusively in the circulation and binds to preformed complexes composed of IGF and IGFBP-3 or IGFBP-5. Due to the size of the ternary complex, approximately 80% of all IGF-I is sequestered in the intravascular compartment. By contrast, the IGFs are predominantly bound to the IGFBPs in binary complexes within tissues (9). Consequently, the IGFBPs create a reservoir of readily available IGF (primarily IGFBP-3 and -5) and control tissue-specific efflux and distribution (primarily IGFBP-1,-2, and -4) (1, 2). Thus, the IGFBPs serve as important determinants of IGF actions and like the IGFs, the IGFBPs have been suggested to play a role in the pathogenesis of atherosclerosis. Of note, in the fetal and adult human heart, IGFBP-3 appears to be expressed at high levels (10), and in the developing rat heart, mRNA for IGFBP-3,−4, and−5 has been demonstrated (11). Importantly, based on the GeneCards^R^ database entries, all IGFBPs can be detected in the normal human heart. Accordingly, several IGFBPs have been suggested as attractive cardiovascular markers, although local vs. systemic effects of IGFBPs in the heart have not been resolved and causal relationships between IGFBP perturbations and the development of atherosclerosis remain to be firmly established (12). While the biomarker potential of growth hormone (GH) or IGF-I in heart failure has been discussed just recently (13), the present review for the first time addresses the biomarker potential of all IGF binding proteins (IGFBPs) in cardiovascular diseases. In order to understand the potential effects of local IGFBP-expression in the heart, the discussion of clinical studies is extended by concepts and hypotheses derived from selected functional studies of IGFBPs in the heart and entire circulatory system. Cardiovascular disease is a group of diseases that includes both the heart and blood vessels, and of which most are caused by atherosclerosis, where the inside of an artery narrows due to the accumulation of an atherosclerotic plaque. Ischemic heart disease [IHD, alternatively coronary artery disease (CAD) or coronary heart disease (CHD)] is the umbrella term for stable and unstable angina, acute myocardial infarction (AMI, commonly known as heart attack), and sudden cardiac death. AMI and unstable angina defines the acute coronary syndrome (ACS). Stroke is the subtype caused by a disruption in the flow of blood to part of the brain either due to blood vessel occlusion (ischemic stroke) or rupture (hemorrhagic stroke). Congestive heart failure is the end stage of several circulatory diseases and characterized by abnormal myocardial function and insufficient ability to maintain blood flow.

On a final note, it should be acknowledged that research in the area is still in its early phases, and larger and more comprehensive investigations are needed to fully assess the biomarker potential of the IGFBPs as well as potential redundancy function within the IGFBP family. Moreover, it is pivotal to appreciate the relation of cause and effect. Most theories derive from findings in cross-sectional studies and such observations should be considered as hypothesis generating as they cannot give evidence of causality. Finally, in all paragraphs to come, it is urged to remember that numerous studies have been performed using assays with undocumented specificity toward the IGFBPs, and our evaluation and interpretation of studies should be seen in the light of that.

## IGFBP-1

Much attention has focused on IGFBP-1 as a partaker in metabolic diseases, as it is negatively regulated by insulin, glucose, and GH (14). In prediabetic patients, IGFBP-1 is reduced, but as the disease progresses, so does pancreatic secretory capacity, resulting in chronic insulin deficiency as well as increased IGFBP-1 levels. Thus, the biomarker potential of circulating IGFBP-1 is largely biased by conditional and age-related insulin or GH-insensitivities. In the heart, IGFBP-1 expression is differentially regulated by insulin (15). In smooth muscle cells, IGFBP-1 expression (Table 1), which is increased by interleukins and TNFalpha, exerts IGF-dependent as well as—independent effects on cell proliferation (16). Because higher concentrations of IGFBP-1 were found in aortic plaques, IGFBP-1 was discussed in the context of plaque stability (16). However, mice overexpressing IGFBP-1 present with reduced blood pressure and increased vascular nitric oxide production, and the overexpression prevents vascular endothelial dysfunction in the mice on high calorie diet [107]. In patients with AMI, significant reductions in serum IGFBP-1 (~40 ng/ml) when compared to healthy subjects (~70 ng/ml) have been demonstrated (20). Likewise, in patients with type 2 diabetes (T2D), decreased circulating concentrations of IGFBP-1 were correlated with cardiovascular risk factors such as low high-density lipoprotein (HDL) cholesterol or high blood pressure (17). These correlations have been confirmed by several consecutive studies (18, 19). However, in patients with heart failure, IGFBP-1 levels have been shown to be increased, although not associated with outcome (21), and in 112 patients with unstable angina, IGFBP-1 levels correlated with ACS disease severity and are higher in patients with multivessel disease than those with single-vessel (22). High circulating IGFBP-1 was also significantly associated with morbidity and cardiovascular mortality in a study including more than 500 diabetic patients with AMI (23). Thus, it was concluded that metabolic control and hepatic insulin resistance are related to fatal events in diabetic CVD patients. Notably, in the lowest or highest IGFBP-1 tertile, IGFBP-1 concentrations ranged between 2 and 24 or 43 and 677 μg/l, respectively (23). In diabetic patients, IGFBP-1 concentrations were associated with those of copeptin, which independently predicted myocardial events, and this could in part explain the prognostic value of IGFBP-1 for heart failure or AMI (56). However, the same association was not found in a more recent study by the same authors (24). On the tissue level, sonography of carotid arteries in type 2 diabetic patients revealed a highly significant negative correlation between IGFBP-1 serum concentrations and thickness of the combined intimal and media compartments (25). Irrespective of diabetes, high IGFBP-1 serum concentrations were correlated with and thus predictive for an increased risk of cardiovascular and coronary heart disease mortality in elderly men (26). Likewise, in survivors of a previous (first) AMI, higher circulating IGFBP-1 concentrations predicted heart failure as demonstrated by a prospective study (27) which included male and female subjects between 45 and 70 years of age. Interestingly, higher IGFBP-1 concentrations were informative for mortality in subjects with no history of heart failure after a follow-up of 8 years. Serum IGFBP-1 concentrations were also significantly increased in patients with critical CAD when compared to patients with less severe CAD (22). In combination with HDL cholesterol, IGFBP-1 serum concentrations were more sensitive and specific for the prediction of CAD (22). Lower IGFBP-1 and IGF-I serum concentrations were associated with an increased risk of IHD later in life or with higher cardiovascular disease mortality in men and women at an age between 51 and 98 years (28). Nevertheless, the authors also concluded that assessment of IGFBP-1 and IGF-I could be used for the identification of adult subjects at an increased risk of fatal IHD as well as for the selection of an appropriate intervention strategy. The reasons underlying the contradictory biomarker information of IGFBP-1 are not directly evident, because the follow-up periods were 9–13 years in one study (28) and 8 years in the later study (27). A possible explanation may be deduced from the fact that the study by Janszky et al. was restricted to the risk of heart failure but not to all-cause mortality or mortality related to cardiovascular disease. In 335 elderly male subjects (70–89 years of age), IGFBP-1 was not associated with increased prevalence of cardiovascular mortality risk (29). Concentrations of IGFBP-1 in the circulation furthermore were not correlated with the prevalence of coronary complications in aged subjects (30). Collectively, these findings suggest that IGFBP-1 may predict future cardiovascular mortality and morbidity, but perhaps more importantly, it may serve as a marker of hyperinsulinemia, which precedes subsequent development of insulin resistance and CVD.

**Table 1:** Biomarker potential of IGFBPs in ischemic heart disease.

**IGFBP-**	**Patient information**	**Biomarker association**	**Method**	**References**
1	Aortic plaques	SMC proliferation (+) plaque stability (+)	mRNA	(16)
1	Diabetic	CV risk factors: insulin (–), blood pressure (–)	RIA	(17, 18)
1	Aged (m: 70–89 y)	CV risk factors (–)	IFA	(19)
1	AMI	IGFBP-1 (–)	IRMA	(20)
1	Heart failure	Heart failure (+)	RIA	(21)
1	CAD	Severity of CAD (+)	ELISA	(22)
1	Diabetic and AMI	Morbidity and CV mortality (+)	RIA	(23)
1	ACS	Copeptin as a marker of AMI (no effect)	RIA	(24)
1	Diabetic	Carotid IMT thickness (+)	IFA	(25)
1	Aged (m: 65–84 y)	CV and CHD mortality (+)	IFA	(26)
1	AMI survivor (45–70 y)	Heart failure (+)	RIA	(27)
1	Healthy (45–70 y)	Heart failure (+)	RIA	(27)
1	CAD	Severity of CAD (+)	ELISA	(22)
1	Aged (51–98 y)	CVD mortality (–)	IRMA	(28)
1	Male (70–89 y)	Cardiovascular mortality risk (no effect)	IFA	(29)
1	Aged	Coronary complications (no effect)	ELISA	(30)
2	Diabetic	Cardiovascular risk factors (–)	RIA	(31)
2	IHD	Death/MI (+)	n.p.	(32)
2	Diabetic and controls	Carotid-femoral pulse wave velocity (–)	IDS-iSys	(33)
2	Aged (≥80 y)	Mortality (+)	RIA	(34)
2	Aged (63–82 y)	Arterial IMT (–)	ELISA	(35)
3	AMI	IGFBP-3 (+)	IRMA	(20)
3	Healthy	IHD later in life (+)	RIA	(36)
3	CHD	IGFBP-3 (+)	EIA	(37)
3	Male	Total cholesterol (+), LDL (+)	IRMA	(38)
3	Hypertension	Carotid atherosclerosis (+)	RIA	(39)
3	Aged (63–82 y)	Plaque instability (+)	RIA	(35)
3	Moderate IHD	Ischemic heart failure (–)	RIA	(40)
3	Adult (40–60 y)	CHD (–)	ELISA	(41)
3	Aged (≥65 y)	Incident coronary events (–)	ELISA	(30)
3	MI	IGFBP-3 (–)	ELISA	(42)
3	CHD	IGFBP-3 (–)	ELISA	(43)
3	Adult/aged (≥45 y)	carotid IMT (–)	CIA	(44)
3	Female (51–68 y)	MI (no effect)	SIA	(45)
3	Subjects (45–79 y)	CAD (no effect)	RIA	(46)
3	Subjects (40–79 y)	Mortality (no effect)	RIA	(47)
3	STEMI/NSTEMI	ACS (no effect)	RIA	(48)
4	Diabetic and controls	Carotid artery remodeling (NT-IGFBP-4) and accelerated atherosclerosis	TR-IFMA	(33)
4	ACS	PAPP-A (+)	n.a./WIB	(49–51)
4	MI-suspected	MACE (NT-/CT-IGFBP-4 +)	IA	(52)
4	CVD	long-term outcome (NT-/CT-IGFBP-4: no effect)	IA	(53)
4	Diabetic	CV mortality (NT-/CT-IGFBP-4 +)	TR-IFMA	(54)
4	STEMI	CV mortality (NT-/CT-IGFBP-4 +)	TR-IFMA	(55)
5	CHD	IGFBP-5 (+)	RIA	(37)
5	CHD and healthy controls	Apo A1 (+) HDL-C (+)	RIA	(37)

*ACS, acute coronary syndrome, AMI, acute myocardial infarction; CAD: coronary artery disease; CHD, coronary heart disease; CIA, chemiluminescence immunoassay; CT-/NT-, carboxyl-/amino-terminal); CV, cardiovascular; CVD, cardiovascular disease; EIA, enzyme immunoassay; H/LDL, high-/low-density lipoprotein; IA, immunoassay; IDS-iSYS, Immunodiagnostic Systems (automated immunoassay analyzer); IFA, immunofluorometric assay; IHD, ischemic heart disease; IMT, intima-media thickness; IRMA, immunoradiometric assay; MACE, major adverse cardiac event; MI, myocardial infarction; n.a., not applicable; n.p., not provided by the authors; N/STEMI, no/elevation of the ST segment; PAPP-A, pregnancy associated plasma protein A; RIA, radioimmunoassay; SIA, 2-step sandwich-type immunoassay; TR-IFMAs, time-resolved immunofluorometric assay; WIB, Western immunoblot*.

## IGFBP-2

IGFBP-2 has been established as a marker of the metabolic syndrome and therefore, it has been suggested that low concentrations of IGFBP-2 could be a useful biomarker for the assessment of cardiovascular risk factors (31). It is the second most abundant binding protein in circulation and is also metabolically regulated, albeit not as rapidly as IGFBP-1. Indeed, IGFBP-2 levels are reduced in obese subjects and in T2D, and low levels associate with elevated fasting glucose, serum triglycerides, and LDL cholesterol (31). As a particular advantage of IGFBP-2 as compared to IGFBP-1, circulating IGFBP-2 concentrations are less prone to post-prandial alterations. This suggests IGFBP-2 to represent a more robust biomarker than IGFBP-1 (31). In a cross-sectional study that included 310 study members at an age between 63 and 82 years, circulating IGFBP-2 concentrations were negatively correlated with arterial intima-media thickness (IMT), whereas IGF-II levels were positively associated with IMT (35). Conversely, IGFBP-2 concentrations in plasma were about 2-fold increased in 273 cases of fatal IHD and a strong association between IGFBP-2 and death/AMI was described (32). In 99 patients with T2D and 99 controls, IGFBP-2 was inversely associated with pulse wave velocity, which is a measure of arterial stiffness and thus, the degree of atherosclerosis (33). However, these cross-sectional studies reflect associations and give no evidence of causality. In a study by Hedbacker et al. (57), the authors demonstrated a direct beneficial effect of IGFBP-2 on cardiovascular risk factors in excessively obese (ob/ob) and diabetic mice. IGFBP-2 overexpression resulted in a 3-fold increase in hepatic insulin sensitivity and a reduction in plasma glucose, liver triglycerides, and hepatic steatosis. As a result, the diabetic phenotype was remedied. The same reduction in plasma glucose was observed upon overexpression of IGFBP-2 in wild-type mice, and there was a trend for reduction in insulin levels in both models. Considering this association between low IGFBP-2 and multiple CVD risk factors, IGFBP-2 may serve as a robust biomarker for the identification of individuals with high cardiovascular risk. However, IGFBP-2 is also a biomarker of mortality in elderly subjects (34), possibly explained by an association between high serum IGFBP-2 and low physical function. Thus, it might be important to also assess the functional relevance of increased circulating IGFBP-2 levels for CVD in the future.

## IGFBP-3

Due to its abundance in the circulation and role as the primary IGF carrier, substantial attention has been paid to IGFBP-3. Surprisingly, little is known about its regulation with regards to cardiovascular disease. In the human heart, IGFBP-3 is expressed throughout lifetime both on mRNA and protein levels and IGFBP-3 mRNA expression in the heart is higher when compared to the liver at fetal as well as adult stages (10). Interestingly, higher IGFBP-3 protein expression was identified by Western immunoblotting in the ischemic as compared to the hypertrophic or dilated heart (10). Intact IGFBP-3 is secreted by cardiac atrial appendage stem cells (CASCs) and thought to be related to the regenerative potential due to cardiac angiogenesis and possibly also to some extent cardiomyogenic differentiation in the ischemic heart as demonstrated also by Western immunoblotting (58). After heart transplantation, expression of IGBP-3 and IGF-I mRNA was quantified in end-stage dilated (*n* = 11) or ischemic hearts (*n* = 12) (59). Compared to healthy control hearts (*n* = 10), expression of IGFBP-3 mRNA was significantly increased in both pathological conditions, whereas IGF-I mRNA was elevated only in the dilated heart (59). Expression of IGFBP-3 was further discussed in the context of hypoxia and a pathophysiological function of the IGF-system was proposed for the heart. In fact, in cultivated H9c2 myocardial cells, hypoxia increased expression of IGFBP-3, which was responsible for the induction of apoptosis due to inhibition of IGF-signaling via protein kinase B (60). However, on the local level, IGFBP-3 exerted antiproliferative effects on the Wnt signaling pathway in cardiac progenitor cells (61). Thereby, the authors demonstrated that the antiproliferative effect of IGFBP-3 depends on an intact IGF-binding site.

### Increased concentrations of IGFBP-3

In contrast to IGFBP-1, IGFBP-3, and IGF-I levels significantly increased after AMI (20). The authors were able to specify a threshold of 137 ng/ml for the serum concentrations of IGF-I: lower concentrations were correlated with a worse prognosis in AMI patients, whereas higher concentrations of IGF-I after AMI were associated with improved functional parameters detected by echo cardiography (e.g., left ventricular mass or ejection fraction) (20). It was suggested that high IGF-I immediately after AMI fueled myocardial remodeling, and that IGFBP-3, which generally reflects total IGF-I levels, increased in parallel. In a prospective study, high IGFBP-3 and low IGF-I serum concentrations in healthy subjects were correlated with an increased risk of developing IHD later in life (36). In partial agreement, higher levels of IGFBP-3 but also higher serum concentrations of IGF-I were described in patients with CHD (37). Under conditions of AMI, an elevation of the ST segment (STEMI) on the electrocardiogram (ECG) is considered to characterize a more severe form of the ACS when compared to patients with no elevation of the ST segment (NSTEMI). In a longitudinal study of 747 White and 544 Black young males over a period of 10 years, an increase of IGFBP-3 serum concentrations over time was associated with significant increases of total cholesterol and LDL cholesterol (38). This study did not confirm previous cross-sectional studies describing associations of IGFBP-3 and IGF-I with hypertension, but rather revealed a link between IGFBP-3 and lipid concentrations in young males. In hypertensive patients, a role of IGFBP-3 in the formation of carotid atherosclerosis was suggested (39). In elderly patients (63–82 years of age), higher circulating concentrations of IGFBP-3 or IGF-I were positively or negatively associated with plaque instability, respectively (35). The authors concluded that IGF-I and IGFBP-3 might represent functional biomarkers for prediction of the risk for plaque rupture or therapeutic targets for stabilization of atherosclerotic plaques. In fact, systemic concentrations of IGFBP-3 and IGF-I were increased after application of the angiotensin-converting enzyme-inhibitor Fosinopril, and the beneficial effects of treatment in IHD were discussed in the context of the IGF-system (62).

### Conditions of decreased circulating IGFBP-3

In addition to IGF-I and IGFBP-3, secretion of GH was also impaired in patients with ischemic heart failure (40). The authors observed patient-individual GH-sensitivity related to the degree of left ventricular dysfunction (40). In male patients between 40 and 60 years of age (41) and in adults of both genders older than 65 years (30), lower concentrations of IGFBP-3 or IGF-I in serum were associated with CHD. In patients with AMI, serum levels of IGFBP-3, IGF-I, and IGF-II were decreased immediately after AMI, but returned to their normal range 1 week after coronary intervention (42). Similarly, in 90 patients with a diagnosed CHD, reduced levels of circulating IGFBP-3 and IGF-I were described (43). The results are in principal agreement with a previous report on reduced IGF-I concentrations in patients with AMI (63). In both studies, serum concentrations of GH were increased under conditions of acute cardiac ischemia. Low concentrations of IGFBP-3 and high circulating levels of IGF-I were further associated with an increased thickness of the intima-media (44).

### Lack of biomarker value for IGFBP-3 concentrations

A prospective study in women at 51–68 years of age revealed no direct relationship between IGFBP-3 or IGF-I and a risk of AMI later in life (45). Systemic effects of IGFBP-3 or IGF-I on the development of CAD were excluded by a prospective study observing more than 1,000 cases and more than 2000 controls over a mean period of 6 years (46). From this study and from an additional meta-analysis assessing 31 single nucleotide polymorphisms present in the *IGF1* and *IGFBP3* genes, the authors concluded that neither circulating IGFBP-3 or IGF-I nor the corresponding genes were causative for the development of CAD in human populations. At a later time point, a single nucleotide polymorphism upstream of the *IGF1* genomic region was identified in patients at an increased risk of developing CAD (64). With respect to IHD, an association between mortality and circulating concentrations of IGFBP-3, IGF-I, or IGF-II was also excluded by a large prospective Japanese study including more than 39,000 subjects between 40 and 79 years of age (47). In a Turkish study including 20 STEMI patients, 10 NSTEMI patients, and 20 healthy controls, IGFBP-3 was not affected by ACS (48). Instead, IGF-I was severely reduced in ACS patients with elevations of the ST segment on the ECG (48). Therefore, IGF-I serum concentrations were suggested to represent potential biomarkers of myocardial necrosis in the STEMI group of ACS patients.

### Age-related biomarker potential of IGFBP-3

Interestingly, age-related discrepancies exist in the IGF- and IGFBP-concentrations after AMI as analyzed by RIA (65). Accordingly, younger subjects (<50 years of age) are characterized by higher absolute concentrations and lower daily variations of circulating IGF-I when compared to more aged subjects (>50 years of age), which may be related to higher regenerative capacities in younger subjects after AMI (65). Age-related differences of IGF-I and IGFBP-3 in the context of differential prognosis after AMI also might indicate an involvement of GH during functional recovery of the infarcted heart. The inclusion of structural information (intact vs. fragmented IGFBPs) could be used in order to assess age-related control of IGF-bioactivity.

## IGFBP-4 and PAPP-A

IGFBP-4, also an abundant IGFBP, has been suggested in numerous studies to be directly involved in the inhibition of atherosclerosis (33, 52, 54, 55, 66, 67). As demonstrated on the level of mRNA and protein, intact IGFBP-4 is produced by a variety of cell types and represents the major IGFBP secreted by myoblasts (68–70). It is considered to attenuate IGF activity in most physiological contexts and inhibits proliferation and differentiation during the transition from myoblasts to myotubes (68). Studies suggest that intact IGFBP-4 modulates cardiac development and cardiomyocyte differentiation (70) and is actively involved in the development of atherosclerosis, with high expression levels in aorta lesional areas (66). Moreover, mice with an overproduction of IGFBP-4 present with smooth muscle hypoplasia (71). IGFBP-4 has a preferential affinity for IGF-II over IGF-I and transports them to peripheral tissues, where proteases cleave IGFBP-4 into low binding-affinity fragments (72–74). The enzyme pregnancy-associated plasma protein-A (PAPP-A) has been identified as the principal, if not only, protease responsible for this IGF-dependent cleavage of IGFBP-4 (75, 76), and PAPP-A levels strongly correlate with the levels of IGFBP-4 proteolytic fragments (54). PAPP-A tethers to cell surfaces, and thus, release of the IGFs occurs in close proximity to the IGF-IR and increases IGF bioavailability primarily at local sites (77). Interestingly, balloon injury of porcine coronary arteries has been shown to massively increase the expression of PAPP-A and the subsequent degradation of IGFBP-4 in the neointimal and medial layers, peaking at 4 weeks after treatment, thus causing possible hyperplasia and coronary restenosis (71). In a study of patients with T2D and healthy controls, IGFBP-4 fragment levels were associated with the normalized wall-index, which is a measure of carotid artery remodeling and accelerated atherosclerosis (33). In murine models, deletion of the PAPP-A gene resulted in an 80% reduction in atherosclerotic area, whereas transgenic overexpression of PAPP-A accelerated plaque progression (66, 78). Inhibition of the PAPP-A substrate binding site with a neutralizing monoclonal PAPP-A antibody caused a 70% reduction in plaque area (79). Importantly, when PAPP-A is deprived of its proteolytic activity, intact IGFBP-4 levels increase and diminish the actions of IGF-I. Collectively, the findings suggest that high PAPP-A and low IGFBP-4 levels may exacerbate the pathophysiological processes underlying plaque development. Under conditions of myocardial damage, PAPP-A concentrations are also increased in the circulation (49). In 2001, Bayes-Genis et al. were the first to demonstrated that PAPP-A was ubiquitously expressed in human eroded atherosclerotic plaques, and serum levels were elevated in patients with ACS (50). This initiated a large interest to study PAPP-A as a candidate marker of plaque burden and CVD. However, it was later revealed that administration of heparin to AMI patients, which is part of the standard initial treatment, results in a rapid increase in PAPP-A concentrations, possibly through a displacement of cell surface attached PAPP-A (51). Instead, Postnikov et al. proposed that quantification of the PAPP-A generated, cleaved amino-terminal (NT) and carboxyl-terminal (CT) fragments of IGFBP-4 could be reflective of PAPP-A enzymatic activity and hereby serve as prognostic biomarkers (12, 52). In patients with symptoms of IHD, both fragments were increased in the case of short-term cardiac events (coronary bypass, AMI, or cardiac death) (52). However, in a follow-up study by the same authors, neither IGFBP-4 fragments nor PAPP-A sufficed to predict the long-term outcome in patients with stable cardiovascular disease (53). The varying results may relate to differences in follow-up period and number of patients and hence, events. Recently, the fragments were shown to prognosticate cardiovascular mortality in 330 type 1 diabetes patients without cardiovascular disease at baseline during 12 years of follow-up (54) and in 656 patients with STEMI followed for 5 years (55). Importantly, it was also verified that the IGFBP-4 fragments, unlike PAPP-A, were unaffected by heparin treatment of coronary patients (80). Interestingly, accumulating evidence argues for multiple IGF-independent functions of IGFBP-4. In IGF-insensitive colorectal cancer cells, recombinant human IGFBP-4 was able to efficiently block colony formation (81), and in P19CL6 or in embryonic stem cells, knockdown of IGFBP-4 was shown to alter cardiomyogenesis and cardiac regeneration (70). The mechanism was mediated through inhibition of canonical Wnt signaling and not through IGF. In mice, an IGFBP-4 variant blocked translocation of beta-catenin to the cell nucleus in the ischemic heart (82). Injection of IGFBP-4/H95P directly after AMI prevented beta-catenin related DNA-damage in cardiomyocytes and reduced infarct size 4 weeks after AMI in the mouse (82). Because IGFBP-4/H95P is lacking the intact IGF-binding domain, the protective effect of mutated IGFBP-4 in the ischemic heart is IGF-independent.

## IGFBP-5

IGFBP-5 is produced and secreted by numerous cell types, including vascular smooth muscle cells, and its expression is increased within atherosclerotic plaques (83). Furthermore, atherosclerotic arteries exhibit staining of IGFBP-5 staining along intimal plaques (84). In a cross-sectional study of 95 male CHD patients and 92 healthy controls, elevations of IGFBP-5 and acid labile subunit (ALS) were measured in serum (37). In addition, IGF-I, IGF-II, IGFBP-3, and ALS were increased under conditions of CHD in the patients (37). In serum, concentrations of IGFBP-5 were significantly correlated with the levels of IGF-I and -II or IGFBP-3 (37). The increased concentrations of IGFBPs including IGFBP-5 and ALS could be causative for the larger amounts of IGF-I and -II in CHD patients (37).

Under ischemic conditions, local expression of IGFBP-5 mRNA, which succeeds the initiation of IGF-II mRNA expression, is thought to terminate cardioprotection exhibited by IGF-II (85–87). In fact, a dual role has been suggested for IGFBP-5, with positive effects on IGF-I-stimulated differentiation of myoblasts *in vitro*, yet inhibitory effects on IGF-II activity (68). Thus, the association of IGFBP-5 with atherosclerosis may indicate a direct stimulatory effect of IGFBP-5 on smooth muscle cell proliferation and plaque formation.

## IGFBP-6

IGFBP-6 exerts both IGF-dependent and -independent effects in various cell types (88). In endomycardial biopsies from 12 patients, IGFBP-6 mRNA expression was increased after explantation of a left ventricular assist device when compared to biopsies sampled during implantation (89). Because other proteins in the IGF-system, including IGF-I, IGF-II, and IGFBP-4, were also increased after reverse remodeling of the heart, it was concluded that local effects of the IGF-system are active during heart recovery (89)—yet, a direct link to IHD remains unestablished. However, increased expression of IGFBP-6 after prolonged hypoxia has been observed in vascular endothelial cells (90). Notably, the *IGFBP6* gene promotor contains several hypoxia response elements, which could explain the increased expression of IGFBP-6 (90). IGFBP-6 levels were also increased after hypoxia in vascular endothelial cells (90). However, since expression of IGFBP-1 was increased as an earlier response to hypoxemic conditions (91, 92) than IGFBP-6, this may be interpreted as an example of molecular job sharing between distinct members of the IGFBP-family.

## Summary and conclusions

So far, clinical studies have demonstrated positive or negative associations and a number of studies have also concluded that the IGFBPs show no prognostic value in the assessment of cardiovascular risk. Certainly, one reason for the controversies present in the literature could be the fact that the IGF system is involved in so many biological processes, and hence, the complex nature and multifunctional properties of the IGFBPs. Firstly, alterations in IGFBP levels may represent a phenomenon caused by the disease *per se*. However, most patients receive medical therapy, which affects several components of the IGF-system and constitutes a significant confounder. The effect of heparin on the IGFBP-4 protease PAPP-A is an example of that. Acute patients often also present with an altered peripheral tissue perfusion, allowing leakage of tissue-localized IGFBPs. Furthermore, IGFBP levels may also fluctuate due to compensatory mechanisms or be affected by age or metabolism. It is well-established that IGFBPs are controlled by both GH and insulin, and under conditions of acute or prolonged illness, altered secretory patterns of GH and insulin can increase or decrease levels of IGFBPs (65, 93). Secondly, assessment of IGFBP biomarker potential highly depends on accurate measurements of the IGFBP. Thus, it is important to know of the factors that can potentially skew these measurements and it is crucial that immunoassays are based on antibodies that recognize all clinically relevant forms of the analyte. Unfortunately, most studies use immunoassays without information on target epitopes, and it is well-known that many assays exhibit cross-reactivity toward structurally similar IGFBPs or are unable to discriminate between various IGFBP forms (80). Since the IGFBPs can be proteolytically degraded by proteases, resulting in circulating IGFBP fragments, it is crucial that the assays can distinguished between intact and fragmented IGFBP. Several proteases have been identified, and although these proteases, like most other members of the IGF system, appear under the strict control of paracrine and endocrine influences, our knowledge on factors that control protease activity and tissue distribution is far from sufficient (12). Additionally, the IGFBPs are subject to post-translational modifications such as phosphorylation and glycosylation and may also be partially truncated or in complex with other proteins. Indeed, it is known that the glycosylation patterns of many proteins are altered in various disease states (94, 95), and it is likely that both proteolysis and post-translational modifications of the IGFBPs are influenced by an acute setting and differs from that of stable patients (96). Thus, the use of analytical systems with no discriminatory potential toward intact or modified IGFBPs represents a major issue in IGFBP-related biomarker research, and we prospectively need to include structural and functional information on the IGFBPs. This issue, which is not restricted to CVC patients, further raises concerns regarding the reliability and consequently, the utility of IGFBPs, and at present, identification of patients with high CVD risk cannot solely rely on IGFBP measurements.

It should be noted that research in the area is still in its early phases, and we advocate that larger and more comprehensive investigations are implemented before evaluating the biomarker potential of the IGFBPs. Investigations so far have mostly been limited to cross-sectional studies and animal studies, and cautious interpretation is necessary, as data should be confirmed by longitudinal and mechanistic studies. Accordingly, broader population studies including more complex settings of pathophysiological conditions in their subjects may come to the conclusion that no associations are present between circulating IGFBPs and cardiovascular parameters. We speculate that the inclusion of patients with a higher degree of overlap with respect to their pathophysiology could improve the assessment of the biomarker potential of the IGFBPs.

Our understanding of the IGFBPs has advanced throughout recent years, and the clear link between the IGFBPs and a number of pathological conditions has preserved the interest in identifying novel biomarkers that may help improve future diagnosis and prognosis. However, studies regarding CVD have been varying and discrepant, and it remains unknown to what extent IGFBP measurements possess clinical value, in particular in the absence of structural and functional information on the IGFBPs. Further understanding of the IGF system in both normal physiology and specifically in CVD is necessary to avoid misinterpretations.

## Author contributions

AH wrote the manuscript and prepared the table; RD revised the manuscript; RH wrote the manuscript.

### Conflict of interest statement

AH is related to Ligandis UG.

The remaining authors declare that the research was conducted in the absence of any commercial or financial relationships that could be construed as a potential conflict of interest.

## Abbreviations

ACS: acute coronary syndrome
ALS: acid labile subunit
AMI: acute myocardial infarction
CAD: coronary artery disease
CASC: cardiac atrial appendage stem cell
CHD: coronary heart disease
CT: carboxyl-terminal
CV: cardiovascular
CVD: cardiovascular disease
ECG: electrocardiogram
GH: growth hormone
HDL: high-density lipoprotein
IGF: insulin-like growth factor
IGFBP: IGF binding protein
IHD: ischemic heart disease
IMT: intima-media thickness
LDL: high-density lipoprotein
MACE: major adverse cardiac events
MI: myocardial infarction
MMP: matrix metalloproteinase
NSTEMI: no elevation of the ST segment
NT: amino-terminal
PAPP-A: pregnancy-associated plasma protein-A
STEMI: elevation of the ST segment
T2D: type 2 diabetes
TNF: tumor necrosis factor.
